# AttentionTTE: a deep learning model for estimated time of arrival

**DOI:** 10.3389/frai.2024.1258086

**Published:** 2024-08-23

**Authors:** Mu Li, Yijun Feng, Xiangdong Wu

**Affiliations:** ^1^School of Computer Science and Engineering, Beihang University, Beijing, China; ^2^Ecole Centrale de Pékin, Beihang University, Beijing, China

**Keywords:** self-attention, TTE, deep learning, time serial data, transformer

## Abstract

Estimating travel time (ETA) for arbitrary paths is crucial in urban intelligent transportation systems. Previous studies primarily focus on constructing complex feature systems for individual road segments or sub-segments, which fail to effectively model the influence of each road segment on others. To address this issue, we propose an end-to-end model, AttentionTTE. It utilizes a self-attention mechanism to capture global spatial correlations and a recurrent neural network to capture temporal dependencies from local spatial correlations. Additionally, a multi-task learning module integrates global spatial correlations and temporal dependencies to estimate the travel time for both the entire path and each local path. We evaluate our model on a large trajectory dataset, and extensive experimental results demonstrate that AttentionTTE achieves state-of-the-art performance compared to other methods.

## 1 Introduction

In intelligent transportation systems, the estimated time of arrival (ETA) is a critical task (Zhang et al., 2011). Accurate travel time estimations facilitate the optimization of vehicle scheduling and path planning, thereby reducing user travel time by predicting the duration from the starting location to the destination based on given departure times and routes. The complexity of road traffic is closely linked to the accuracy of ETA predictions, making it challenging to forecast travel times under intricate road conditions (Oh et al., 2018).

Various methodologies have been explored for ETA tasks, including the use of AutoRegressive Integrated Moving Average (ARIMA) models (Billings and Yang, 2006). Deep learning approaches, such as Monte Carlo Tree (MCT)-TTE (Liu et al., 2022) and DeepTTE (Wang D. et al., 2018), have significantly improved prediction capabilities by modeling complex spatial correlations and temporal dependencies within trajectories. Additionally, CoDriver (Sun et al., 2020), which is based on the wide–deep–recurrent (WDR) framework (Wang Z. et al., 2018), incorporates an auxiliary task of learning driving styles to integrate driver information, thereby enhancing prediction accuracy. Despite these advancements, there remains a need for improved extraction of spatial correlations and temporal dependencies.

**Figure 1:**
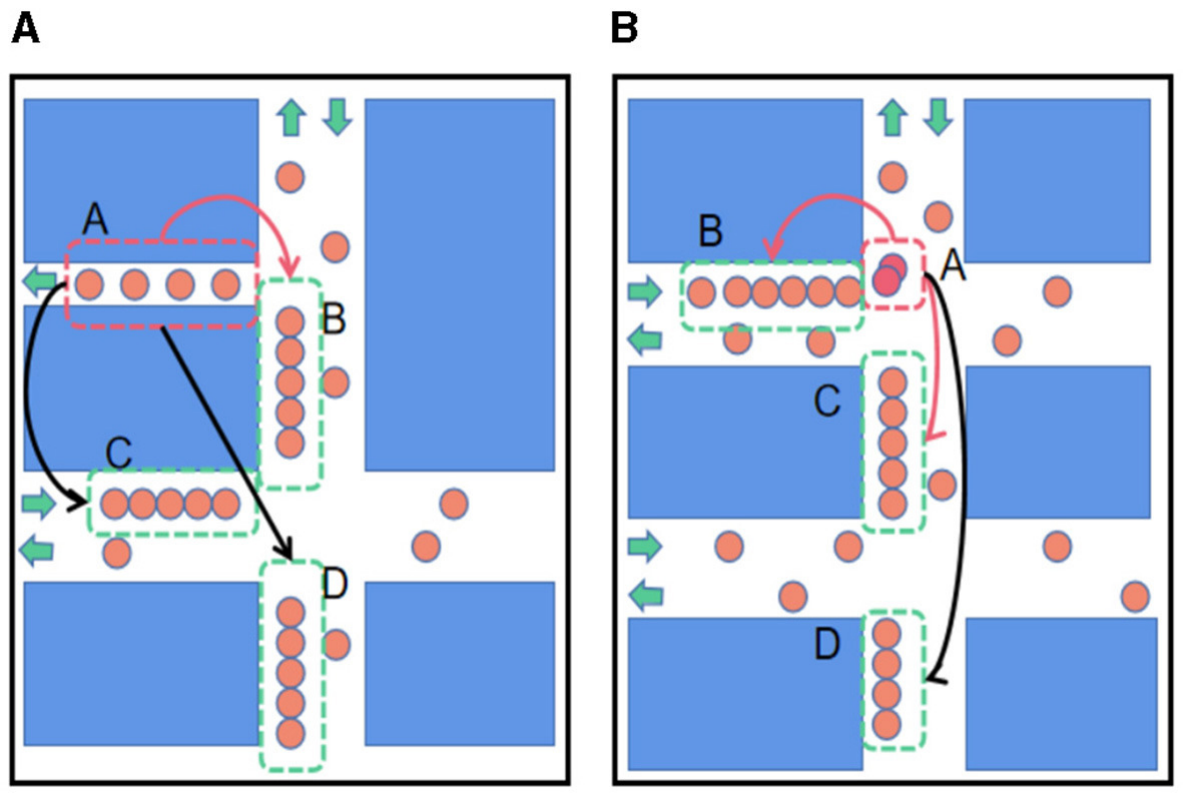
Correlation between sub-segments. In the picture, orange circles represent moving vehicles; the closer the orange circles are, the more congested the traffic. The red circles indicate the cars involved in the accident, and the green arrow indicates the car's direction of travel. The red dotted boxes represent special sub-segments (unusual spatial features or abnormal traffic conditions), and the green ones represent the affected sub-segments. The long arrows represent the impact relationship, where black arrows indicate the impact on the farther sub-segments and red arrows indicate the effect on adjacent sub-segments. **(A)** The influence of spatial feature. **(B)** The influence of abnormal traffic conditions.

Traffic conditions are influenced by a combination of spatial correlations, temporal dependencies, and external information. For instance, a sub-segment with unique spatial features, such as a single one-way lane, can affect upstream and downstream sub-segments if congestion occurs, as depicted in Figures 1A, B. Accurate ETA predictions necessitate consideration of both local and global spatial correlations, temporal dependencies, and external factors such as weather and driver information. Previous research has attempted to integrate external information with each road segment or historical trajectory point to enhance prediction accuracy (Wang D. et al., 2018; Song et al., 2020). However, these methods often fail to account for temporal variations and the interactions between different external factors.

In this study, we proposed an end-to-end deep learning-based arrival time prediction model, termed AttentionTTE, to address these challenges. Our contributions are as follows:

1. An adjusted self-attention mechanism that effectively captures correlations between nodes within the same sequence, highlights the most influential trajectory points, and integrates external information that may affect prediction results.
2. A spatial correlation and temporal dependencies extraction module that learns spatial correlations and temporal dependencies from historical trajectory sequences.
3. A novel external information fusion method, the weighted attribute fusion module, assesses the impact of various attributes (e.g., driver information) on prediction results in different scenarios.
4. An innovative weighting method for collective prediction, leveraging a self-attention matrix to accurately identify the contribution of each local trajectory segment to the overall travel time estimation of the global trajectory.

## 2 Related work

### 2.1 Methods of ETA tasks

Initial ETA research focused on mathematical models like ARIMA for time series forecasting. Though effective, ARIMA struggles with nonlinear data, leading researchers to explore machine learning methods such as Gradient Boosting Decision Trees (GBDT) (Guin, 2006; Zhang and Haghani, 2015). While GBDT scales well, it faces challenges with complex datasets and external information.

Deep learning introduced models from NLP and time series prediction, such as LSTM and RNN (Duan et al., 2016), proving effective for GPS-based travel time prediction. Hybrid models like CNN-RNN captured spatial and temporal correlations, with notable implementations like Yibin Shen's CNN-LSTM for sparse data (Shen et al., 2019) and Dong Wang's end-to-end hybrid model improving accuracy through external information fusion (Wang D. et al., 2018). However, these methods still lack in modeling distant sub-segment correlations and external information interactions.

Further advancements include the WDR model, which views travel time prediction as a regression problem, and the Deep TTE model, which uses geographic convolution and multi-task learning to balance global and local predictions. Despite their accuracy, these models still have limitations, highlighting the need for an ETA method that models long trajectory connections without extensive pre-training and considers external information interactions (Liu et al., 2022).

### 2.2 Individual and collective

ETA task methods based on deep learning can be categorized into individual and collective predictions. Individual prediction methods, such as those proposed by Qiu et al. (2019), divide a complete trajectory into multiple road segment blocks or partial paths based on criteria such as the length of the actual road segment. These methods predict the travel time for each sub-segment sequentially and then aggregate the results to determine the travel time for the entire trajectory. While individual predictions often achieve higher accuracy for each sub-segment, they struggle to account for complex traffic conditions, such as intersections of various road sections, leading to error accumulation and lower accuracy for complete trajectories. On the other hand, collective prediction methods, such as those proposed by Jenelius and Koutsopoulos (2013), predict the travel time for the entire trajectory in one step. Although collective prediction can address issues such as error accumulation inherent in individual predictions, it suffers from reduced confidence when predicting travel times for longer trajectories due to the relatively smaller number of such trajectories available for training (Wang D. et al., 2018). As a result, collective predictions tend to be less accurate for longer travel trajectories.

## 3 Preliminary

In this part, we will give the definition of the problem and some models and techniques related to deep learning.

### 3.1 Problem definition

In this section, we will give some definitions.

#### 3.1.1 Historical trajectory

The historical trajectory is the sampling result of the historical driving trajectory of each driver. We define a historical trajectory sequence P, containing |T| consecutive historical GPS coordinate points, i.e., P ={ p_1_, ... , p_|T|_}. Each GPS coordinate point contains a longitude (p_i_ .lng), a latitude (p_i_ .lat), and a timestamp (p_i_ .ts). For each trajectory, we will record the driver number of the current trajectory, the start time of the journey, the start date of the week, and the current weather conditions.

#### 3.1.2 Travel time prediction

In the training phase, our model learns how to predict the travel time of a given historical trajectory by learning spatio-temporal features from the historical trajectory defined above and the corresponding external information. In the test phase, for each given trajectory, we predict the travel time of the entire trip from the starting point to the endpoint.

#### 3.1.3 Individual prediction

Individual prediction divides a complete trajectory into multiple road segment blocks according to conditions, such as the length of the actual road segment. It predicts the travel time of each sub-segment. In our task, we will split the full trajectory path according to the size of the 1D convolution kernel in the local spatial correlation extraction module.

#### 3.1.4 Collective prediction

The collective prediction directly predicts the travel time of the complete trajectory. We predict travel times for whole trajectories in our task by generating a fixed-length feature vector.

#### 3.1.5 Spatial correlation and temporal dependencies

In a GPS trajectory dataset, the spatial correlation of a trajectory refers to the spatial characteristics of the different areas through which the trajectory passes in real-world geography. Temporal dependencies refer to the time series characteristics of the trajectory.

Local spatial correlation is the spatial correlation between each sub-segment in a complete trajectory and its adjacent sub-track segments; that is, the spatial characteristics of each sub-segment are determined by itself and its adjoining suborbital segments.

Global spatial correlation is the spatial correlation of each sub-segment in an entire trajectory to any sub-segment within the same trajectory; that is, the spatial characteristics of each sub-trajectory segment are determined by all suborbital segments of the complete trajectory together. Based on the local spatial correlation, it further considers the potential spatial correlation between the distant sub-trajectory segment on the trajectory.

### 3.2 Relevant knowledge

In this section, we give a brief introduction to some of the deep learning models and techniques used in our method.

#### 3.2.1 One-dimensional convolutional neural network

The convolutional neural network (Chua and Roska, 1993) is a deep neural network with a convolutional structure. The convolutional structure can reduce the amount of memory occupied by the deep network and the number of parameters of the network and alleviate the overfitting problem of the model. A typical convolutional neural network consists of an input layer, a convolution layer, a down-sampling layer (also called a pooling layer), a fully connected layer, and an output layer. The one-dimensional convolutional neural network (Zhang et al., 2019; Livieris et al., 2020) applies a convolutional neural network in one-dimensional data. Generally speaking, a one-dimensional convolution kernel is usually used to learn the features of text data or time series data because it only convolutes the width but not the height.

#### 3.2.2 Long-short-term memory

Long–short term memory (LSTM) (Hochreiter and Schmidhuber, 1997; Yu et al., 2019) is a kind of recurrent neural network which is widely used in time series and natural language processing. The ingenuity of LSTM lies in that by adding an input gate, forgetting gate, and output gate, when processing the current time step of a sequence, the hidden state of the sequence is dynamically updated according to the current input and the “memory part” (a part of last times hidden state) passed in before. The content of the hidden state is properly “forgotten” by the forgetting gate so that the weight of the self-loop is changed. When the parameters of the model are fixed, the integral scales at different times can be dynamically changed, thus avoiding the problem of gradient disappearance or gradient expansion (Zhu et al., 2015).

#### 3.2.3 Self-attention

Self-attention is one of the attention mechanisms (Vaswani et al., 2017), and it is also a kind of network configuration. Self-attention can make the model better learn the relationship between each node in the same sequence and improve the prediction accuracy by establishing features for the relationship. In general, self-attention was used in natural language processing because of its excellent feature extraction ability for long sequence data.

The self-attention mechanism calculates the attention weight of each sequence fragment by calculating the feature matrix of the sequence. For sequences represented *X* = (*x*_1_, *x*_2_ ... *x*_n_), where $x_{i}\in R^{d_{model}\times 1}$, the weights can be generated by three learnable matrices: *W*^*Q*^, *W*^*K*^, and *W*^*V*^, which generate the three matrices of the sequence *X*, respectively: query matrix, key matrix, and value matrix (as shown in Equations 1–3).


(1) $$\begin{array}{l} Q=W^{Q}\cdot X \end{array}$$

(2) $$\begin{array}{l} K=W^{K}\cdot X \end{array}$$

(3) $$\begin{array}{l} V=W^{V}\cdot X \end{array}$$

where $W^{Q}\in R^{d_{k}\times d_{model}},W^{K}\in R^{d_{k}\times d_{model}},W^{V}\in R^{d_{v}\times d_{model}}$.

The query matrix of each node in the same sequence is multiplied by the key matrix of all other nodes. The result is mapped non-linearly between 0 and 1 through the softmax activation function to obtain the weight assignment matrix. The weight distribution is multiplied by the value matrix to get the attention value *Attention*_*i*_ corresponding to the *i*th node in the attention matrix of the entire sequence (as shown in Equation 4).


(4) $$\begin{array}{l} Attention\left(Q,K,V\right)=softmax\left(\frac{Q^{T}\;\cdot K}{\sqrt{d_{k}}}\right)\cdot V^{T} \end{array}$$

The characteristic of the self-attention mechanism (Shaw et al., 2018) is that the operation object in the weighting process and the weight generation process is the sequence itself, that is, the sequence itself generates the weight involved in the weighted summation in the attention calculation process. Therefore, in the attention matrix *Attention*, the internal relationship between each node in the same sequence is described.

## 4 Model architecture

In this section, we will describe our proposed model the AttentionTTE. AttentionTTE is mainly composed of four parts: (1) the weighted attribute fusion module is used to process the external information (such as driver number and weather) of the current trajectory. (2) The local spatial correlation extraction module is used for mapping the GPS coordinate into the vector space, extracting the local spatial correlation, and generating a feature mapping for the subsequent process. (3) The spatial correlations and temporal dependencies extraction module is the main module of the model, which is mainly used to learn the temporal dependencies of the trajectory, the global spatial correlation of the trajectory, and the fusion of external information items to the trajectory. (4) Multi-task learning module, which is mainly used to predict the travel time of the provided trajectory and balance the individual prediction and collective prediction.

### 4.1 Weighted attribute fusion module

As mentioned in this article, the travel time of historical trajectories depends not only on the coordinate distribution and length of the trajectories in the geographical space but also on various external factors such as drivers, weather conditions, and departure time. These external factors, as attributes for each trajectory, influence the temporal and spatial features, thus affecting the prediction of travel time. Therefore, it is necessary to appropriately consider the impact of external information on temporal and spatial features.

In previous work, most studies used word embedding techniques to incorporate attribute information into trajectories (Gal and Ghahramani, 2016; Wang D. et al., 2018). They embedded attribute information that can influence the travel time into feature vectors and fused them with the feature vectors of the trajectory. Specifically, researchers identified four types of values, including driver ID, weather conditions, date, and departure time, as the attributes. Since these four types of values cannot be directly input into neural networks, they used word embedding techniques (Wang D. et al., 2018) to transform them into low-dimensional vectors.

First, driver ID, weather conditions, date, and departure time are considered as four distinct categorical variables *V*. Among them, departure time is divided into 86,400 categories with intervals of 1 min to cover the entire day. Then, word embedding techniques project each value *v* belonging to the *i*-th original categorical variable, *v* ∈ *V*_*i*_, into the vector space $R^{1\times E_{i}}$ (where *E*_*i*_is the dimensionality of the embedding for the *i*-th categorical variable in the feature vector). Specifically, each original categorical variable value, which has been one-hot encoded, is multiplied by a series of trainable matrices *W*_*i*_ with the same dimensionality to obtain low-dimensional vectors corresponding to each attribute's information. Each matrix *W*_*i*_, $W_{i}\in R^{N_{i}\times E_{i}}$, where *N*_*i*_ represents the number of categories for the i-th original categorical variable *V*_*i*_. Compared to using only one-hot encoding for original categorical variables, word embedding can identify each type of value with fewer dimensions when there is a large number of categories, making computations more efficient. Additionally, in an ideal scenario, after word embedding encoding, similar values of the same type are distributed adjacently in the embedding space, effectively capturing relationships between each type of value.

This approach has limitations. When concatenating and transferring the feature matrices generated by the embedding method for each attribute to the model, each attribute is treated as independent and assumes equal importance in influencing the prediction results. Previous studies primarily considered the impact of attribute information on temporal and spatial features but did not account for the varying degrees of influence that each attribute may have on trajectories under different circumstances. For instance, in real life, weather conditions can negatively affect a driver's mood, and experienced drivers may drive similarly to less experienced drivers in adverse weather (Mikolov et al., 2013a,b).

In such scenarios, the primary factor affecting travel time would be the current weather conditions rather than the driver's personal attributes. This indicates that when using attribute information to improve model performance, the interactions between these pieces of information need to be considered to identify which attribute plays a major role in travel time prediction under specific circumstances. Therefore, exploring more detailed methods to fully utilize attribute information and maximize model performance is essential.

To address this issue, this article proposes a weighted fusion of attribute modules. This module maps all attribute information values to the same-dimensional feature space and dynamically constructs attribute features based on the contribution of each attribute value. Specifically, each attribute is mapped to a low-dimensional space using word embedding techniques, and its vector representation is calculated. Next, a method based on the L2 norm is employed to calculate weights for each attribute. These weights represent the degree of influence that each piece of information has on the final vector. By multiplying the weight of each attribute by its vector representation, weighted attribute features are obtained. Finally, these weighted features are integrated with other model features to achieve accurate predictions of the target variable.

First, we use the embedding technique to transform all attributes into the feature space of the same dimension *E* to obtain the embedded external information as attributes of the trajectory *Attr* as shown in Equation 5.


(5) $$\begin{array}{l} \begin{array}{c} Attr_{V_{i}}=W_{i}^{T}\cdot V_{i}\\ \; \end{array} \end{array}$$

where $Attr_{V_{i}}\in R^{E\times \;1}$.

Second, we get the embedded attributes of each attribute under the current external environmental conditions by a learnable matrix *W*^*env*^ ∈ *R*^*E*×*E*^ as shown in Equation 6.


(6) $$\begin{array}{l} Attr_{V_{i}}^{env}=W^{env}\cdot Attr_{V_{i}} \end{array}$$

Then, to obtain the corresponding weight of each attribute, we calculate the second norm (L2-norm) of each embedded attribute. The L2-norm of each embedded attribute is the modulus value of each attribute in the feature space. The weight of each attribute is obtained by calculating the proportion of its modulus value relative to the total modulus values. In this context, mod\text{mod}mod represents the L2-norm of all attribute vectors, and *mod* represents the L2-norm of each individual attribute vector as shown in Equation 7.


(7) $$\begin{array}{l} mod=\sqrt{|Attr_{V_{1}}^{env}{|}^{2}+|Attr_{V_{2}}^{env}{|}^{2}+\ldots +|Attr_{V_{n}}^{env}{|}^{2}} \end{array}$$

The weight corresponding to each attribute $\alpha_{i}^{Attr}$ is as shown in Equation 8.


(8) $$\begin{array}{l} \alpha_{i}^{Attr}=\frac{mod_{i}}{mod} \end{array}$$

where *mod*_*i*_ is the L2-norm of each attribute vector.

Finally, we weigh and sum the embedded attributes to get the final embedded attributes as shown in Equation 9.


(9) $$\begin{array}{l} attr=\alpha_{1}^{Attr}\cdot Attr_{V_{1}}+\ldots +\alpha_{n}^{Attr}\cdot Attr_{V_{n}} \end{array}$$

Compared with directly fusing each attribute value by embedding coding, the attribute fusion module based on weighting can better learn the difference of the influence of each attribute on the target path at different times and under different conditions.

### 4.2 Local spatial correlation extraction module

Referring to the correlation deep learning method, we use a neural network based on a one-dimensional convolution kernel as our local spatial feature extraction module (Chua and Roska, 1993) to learn the local spatial correlation of several adjacent trajectory points of a historical trajectory. Recall from the previous definition that each historical trajectory consists of a continuous set of GPS trajectory points (containing latitude and longitude information and a sampling timestamp), P = {p_1_, ... , p_|T|_}, and *p*_*i*_ contains longitude (*p*.*lng*), latitude (*p*_*i*_.*lat*), and timestamp (*p*_*i*_.*ts*). The longitude and latitude information in each trajectory point contains the local spatial features and correlation around the trajectory point. We used the local geographic convolutional neural network, which is based on a one-dimensional convolution kernel, to learn the local spatial correlation of each trajectory point and its surroundings.

By stitching the latitude and longitude coordinate values of each trajectory point, we process the raw data into *p*_*i*_ that the neural network can process as shown in Equation 10.


(10) $$\begin{array}{l} p_{i}=p_{i}.lng⚬p_{i}.lat \end{array}$$

Here ⚬ represents the splicing operation of latitude and longitude, and $p_{i}\in R^{2\times 1}$, *p*_*i*_ ∈ *traj, traj* = {*p*_1_, *p*_2_…*p*_*T*_ }.

For each GPS trajectory point *p*_*i*_, it is first converted into a lower-dimensional vector by a learnable matrix *W*_*local*_, $W_{local}\in R^{C\times 2}$, that is *loc*_*i*_ as shown in Equation 11.


(11) $$\begin{array}{l} loc_{i}=\tanh\left(W_{local}\cdot \left(p_{i}\right)\right) \end{array}$$

Therefore, the output sequence *loc* (*loc* ∈ *R*^*C*×*T*^, *loc* = {*loc*_1_, *loc*_2_…*loc*_*T*_}) is a sequence feature of length *T* in the vector space *R*^1 × *C*^, where the *C* channels of each node contain the spatial feature and correlation information of this node.

Second, a one-dimensional convolutional neural network with a parameter size of ${W_{conv}\in R}^{k\;\times \;C}$ (*k* is the size of the convolution kernel, usually an odd number) is used to convolve each GPS trajectory point and its adjacent trajectory points. The local spatial correlation of every *k* adjacent trajectory point is extracted by the convolution operation as shown in Equation 12.


(12) $$\begin{array}{l} path_{local}=relu\left(W_{conv}\;*\;\left(loc_{i:i+k-1}\right)+b\right) \end{array}$$

where $path_{local}\in R^{C\times \left(T-k+1\right)}$, *path*_*local*_*i*__ is the trajectory segment of *loc*_*i*_ to *loc*_*i*+*k*−1_, *b* is bias, ^*^ is a convolution operation.

Then, we append a column to the previously obtained *path*_*local*_, converting the *path*_*local*_ from *R*^*C*×(*T*−*k*+1)^ to *R*^(*C*+1) × (*T*−*k*+1)^, and we get $path_{local}\in R^{\left(C+1\right)\times \left(T-k+1\right)}$. The *i*-th element of the new appended column is the distance of the *i*-th local path. i.e., $\overset{i+k-1}{\underset{j=i+1}{\sum }}Dis\left(p_{j-1},p_{j}\right)$, to mark the order of the whole sequence to facilitate the extraction of global spatial correlation by the self-attention mechanism (Vaswani et al., 2017).

### 4.3 Spatial correlations and temporal dependencies extraction module

In this section, we mainly introduce the main structure of our model, spatial correlations and temporal dependencies extraction module. This module is mainly comprised of two parts, the global spatial correlations extraction module and the temporal dependencies extraction module. Based on the local spatial correlation extraction result, the global spatial correlations extraction module is used to extract global spatial features from the historical trajectory. Finally, we obtained a hidden state of the travel time by cleverly stacking the spatial and temporal features above the two modules.

#### 4.3.1 Global spatial correlation extraction module

**Figure 2:**
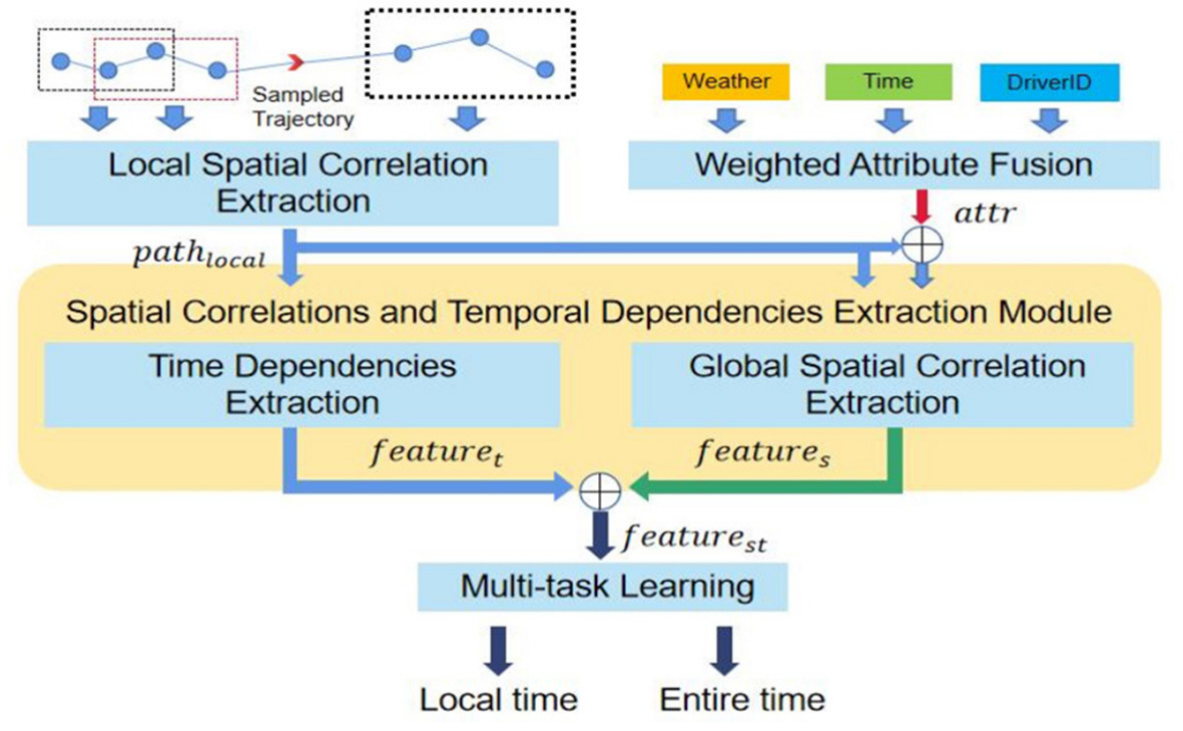
The main structure of the AttentionTTE.

We first propose the global spatial correlation extraction module as shown in Figure 2. This module is an improvement of the self-attention mechanism, enabling it to better learn the potential relationships among nodes within the same sequence in time series data. It dynamically fuses the influence of external information on the sequence. Initially, this module extracts the relevance among all nodes of a local spatial feature sequence using a simple self-attention mechanism. Then, the key trajectory points that have the most significant impact on a sequence's global spatial correlations are identified through the attention weight distribution matrix. Finally, the global spatial correlation is obtained by dynamically fusing the external information.

There is a new sequence of length *T*−*k*+1 containing a local spatial correlation path local, calculated in Equation 12 by the local spatial correlation extraction module. It is worth noting that spatial feature extraction of the trajectory would have been completed if we had followed the traditional method of previous jobs (Wang D. et al., 2018). However, the spatial correlation of a trajectory depends on the spatial features of all trajectory points on the trajectory, and the method obtains the local spatial correlation per *k* trajectory point. It does not consider the global spatial correlation between sub-segments in the complete trajectory. To deal with this problem, we use the self-attention mechanism to learn the sequence containing the local spatial feature and correlation. The self-attention mechanism has a prominent role in processing the relationship between nodes in a complete sequence and has been partially applied in traffic prediction (Cai et al., 2020; Xu et al., 2020). In NLP and other fields, the feature extraction of keywords or nodes in a piece of text or sequence is mainly completed by increasing the dimension of the feature vector of each word or node in the text or sequence and increasing the number of layers of the self-attention mechanism (Vaswani et al., 2017). Still, this process will cause the model parameters to be too large. In the research process of this article, we found that it is difficult for the model to converge after assigning a higher dimension to each trajectory point in the GPS trajectory dataset and using the self-attention mechanism with a higher number of layers. When the feature dimension of each node is small, the feature extraction ability of the self-attention mechanism on the key node decreases. It is not sufficient to use the self-attention mechanism directly in this task.

**Figure 3:**
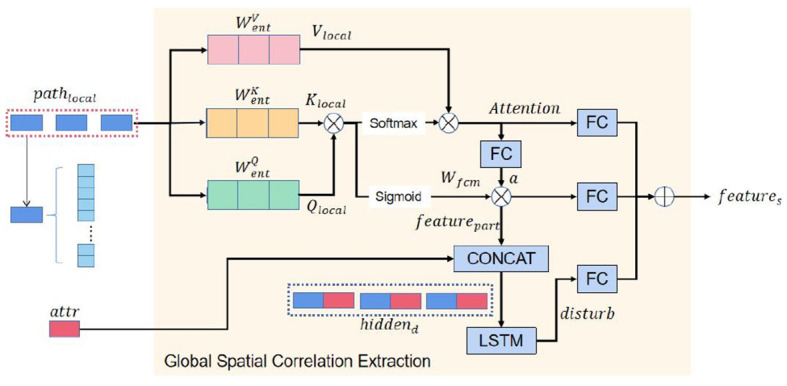
The self-attention of spatial correlations extraction module.

As shown in Figure 3 we use three different learnable matrices ($W_{ent}^{Q}\in R^{d_{k}\times \left(C+1\right)},W_{ent}^{K}\in R^{d_{k}\times \left(C+1\right)},W_{ent}^{V}\in R^{d_{v}\times \left(C+1\right)}$) to multiply each node of the sequence to obtain the query matrix, the key matrix, and the value matrix of the global spatial correlation of the node. In general, to enhance the learning ability of the self-attention mechanism on the relationship between each node of a sequence, we can also use a one-dimensional convolutional neural network with a convolution kernel size of 1 to generate the above feature matrix (Karpov et al., 2020). Compared with the original method, in this task, the self-attention mechanism of using a one-dimensional convolutional neural network to generate *Q, K*, and *V* matrices can be reduced by an average of 0.26% using mean percentage absolute error (MAPE) as the loss function. Taking the node *i* of a trajectory sequence as an example, the calculation process is as shown in Equations 13–15.


(13) $$\begin{array}{l} Q_{local}=W_{ent}^{Q}\cdot path_{local} \end{array}$$

(14) $$\begin{array}{l} K_{local}=W_{ent}^{K}\cdot path_{local} \end{array}$$

(15) $$\begin{array}{l} V_{local}=W_{ent}^{V}\cdot path_{local} \end{array}$$

The query matrix *Q*_*local*_ of each node in the sequence is multiplied by the key value matrix *K*_*local*_ of all other nodes and multiplied by the value matrix *V*_*local*_ by the softmax activation function to obtain the attention weight distribution matrix *Attention*_*local*_ (as shown in Equation 16).


(16) $$\begin{array}{l} Attention=softmax\left(\frac{Q_{local}^{T}\;\cdot K_{local}}{\sqrt{d_{k}}}\right)\cdot V_{local}^{T} \end{array}$$

To extract the influence of critical sub-segments in a trajectory sequence (such as road segments with traffic lights) on other sub-segments, we map the relationship between different sub-segments of the same trajectory to intervals [0,1]. The closer the relationship value between sub-segments is to 1, the stronger the correlation between sub-segments. We achieve this by generating a fuzzy attention allocation matrix using the sigmoid activation function (Kosko, 1986; Felix et al., 2019).

We activate each vertex *Attention*_*i*_ of *Attention* that contains the global spatial correlations of trajectories to state *a*_*i*_ by the activation function tanh and a learnable matrix *W*_*a*_, $W_{a}\in R^{C\times d_{v}}$ as shown in Equation 17.


(17) $$\begin{array}{l} a_{i}=\tanh\left(W_{a}\cdot Attention_{i}^{T}\right) \end{array}$$

Subsequently, we use the sigmoid activation function (Homenda and Jastrzebska, 2019; Wang et al., 2020), query matrix *Q*_*local*_, and key matrix *K*_*local*_ to generate a fuzzy attention allocation matrix *W*_*fuzzy*_ and multiply it with the trajectory state *a* of learning critical node features on the trajectory sequence, and get the *feature*_*part*_ as shown in Equations 18, 19.


(18) $$\begin{array}{l} W_{fuzzy}=sigmoid\left(Q^{T}\cdot K\right) \end{array}$$

(19) $$\begin{array}{l} feature_{part}=W_{fuzzy}\cdot a^{T} \end{array}$$

where, *a* = (*a*_0_, *a*_1_…*a*_*T*−*k*+1_ ).

Usually, the influence of external information on the predicted outcome will change with the duration of driving time (e.g., the driver is tired due to the extended driving time, and the same driver will have different driving styles in the fatigue and normal driving state). This change is often difficult to quantify, but providing an accurate prediction must consider this problem.

To simulate the influence of external information on the prediction results with driving time, the model inputs it into an LSTM to obtain the output of the perturbation term. As each node in the sequence is processed, the LSTM dynamically fuses external information. The advantage of this method is that it can capture the trend and law of the degree of influence of external information on the results with time to better reflect the current external environment information and improve the prediction ability and generalization ability of the model. For example, in traffic forecasting, the dynamic fusion of external information based on weather forecasts, time, and other factors can more accurately predict road congestion and improve the accuracy of traffic forecasting.

To dynamically fuse external information, we concatenate *attr* , *attr* ∈ *R*^1 × *E*^ with each node in the *feature*_*part*_, $feature_{part}\in R^{\left(T-k+1\right)\times C}$ sequence to get the distractor input *hidden*_*d*_, $hidden_{d}\in R^{\left(T-k+1\right)\times \left(C+E\;\right)}$.

In the real world, external information changes over time, such as the driver's driving style, weather conditions, and other information changes over time. To model the process of external information changing with the duration of driving time, we pass *hidden*_*d*_ into a recurrent neural network (recall from the previous description that LSTM is usually very effective in dealing with time series problems) to get the output of the disturbance term. In dealing with each node in the sequence, the recurrent neural network will dynamically fuse external information as shown in Equation 20.


(20) $$\begin{array}{l} disturb=LSTM\left(hidden_{d}\right) \end{array}$$

*disturb* is the feature vector of external information changed with the path, where *disturb* ∈ *R*^(*T*−*k*+1) × *C*^.

Finally, we fuse the above three feature hidden states by three learnable matrices *W*_*fs*_, *W*_*at*_, *W*_*fd*_, to obtain the hidden state *feature*_*s*_ with captured spatial correlations as shown in Equation 21.


(21) $$\begin{array}{l} feature_{s}=W_{fs}\cdot tanh\left(feature_{part}^{T}\right)\\ \;+W_{at}\cdot tanh\left(Attention^{T}\right)\\ \;+W_{fd}\cdot tanh\left(disturb^{T}\right) \end{array}$$

where $feature_{s}\in R^{C\times \left(T-k+1\;\right)}$.

#### 4.3.2 Time dependencies extraction module

We introduce recurrent neural networks into our model to extract the temporal dependencies of historical trajectories. The time dependence is contained in the sequence of individuals of each trajectory segment in a complete trajectory sequence, that is, held in the *path*_*local*_. The recurrent neural network is a deep learning model widely used to capture the dependencies of time series (Duan et al., 2016).

We will extract the time dependencies from the sequence *path*_*local*_ processed by the local spatial correlations extraction module by two layers of connected LSTM to obtain the sequence *feature*_*t*_, with extracted time dependence as shown in Equation 22.


(22) $$\begin{array}{l} feature_{t}=LSTM\left(path_{local}\right) \end{array}$$

We fuse the spatial correlation and temporal dependence into the hidden state *feature*_*st*_ by two learnable matrices as shown in Equation 23:


(23) $$\begin{array}{l} feature_{st}=W_{s}\cdot feature_{s}+W_{t}\cdot feature_{t} \end{array}$$

### 4.4 Multi-task learning module

Finally, we introduce the multi-task learning module. Previous studies on arrival time prediction primarily divided the task into individual and collective predictions. Individual prediction involves dividing a historical trajectory into multiple sub-historical segments (Yang et al., 2013; Wang D. et al., 2018), predicting each segment, and summing the results. Collective prediction directly predicts the travel time of the entire historical trajectory. Generally, individual predictions are more accurate but fail to model the complex traffic environment of the entire trajectory, such as the impact of signal lights and intersections, making them less accurate for longer sequences. Therefore, an ideal model should combine the accuracy of individual predictions with the ability to model complex traffic states in collective predictions. In the experimental phase, we train the model by combining individual and global predictions to improve overall accuracy (Wang D. et al., 2018; Zhang and Yang, 2018).

In the individual prediction stage, we review the previous content. We can make the hidden states sequence *feature*_*st*_ = *hidden* = *h*_1_, *h*_2_.., *h*_*T*−*k*+1_, where each *h*_*i*_ represents a spatial-temporal feature of a local trajectory. We construct an individual prediction unit with two layers of fully connected neural networks. We obtain *T*−*k*+1 scalars, where *t*_*i*_ representing the time of passage by each local trajectory.

Because the feature sequence *feature*_*st*_ is still a hidden state of length *T*−*k*+1, which varies depending on the length of the initial trajectory, we need to convert it to a fixed-length hidden state before making a collective prediction. The commonly used method is to sum and average the *T*−*k*+1 hidden states, which is the mean polling method. However, this method can not effectively determine the contribution of the spatial–temporal features of each local trajectory to the overall trajectory.

Reviewing the content of the self-attention mechanism part, the result is that the hidden state of the sequence processed by the self-attention mechanism contains the correlation between the nodes on the sequence. We calculate the L2-norm value of the hidden state of each node to generate the contribution of the hidden state of each node (*feature*_*st*_) to the entire hidden state (*feature*_*seq*_). We process the sequence *Attention* obtained by the global spatial feature extraction module and calculate the vector modulus in the space *R*^*C*×1^ for each node in the sequence to obtain the modulus sequence *mod, mod* ∈ *R*^1 × (*T*−*k*+1)^. We generate the weight α_*i*_ according to the proportion of the magnitude of the modulus of each node in the modulus sequence as shown in Equation 24.


(24) $$\begin{array}{l} \alpha_{i}=\frac{mod_{i}}{\sum_{j}^{T-k+1}mod_{j}} \end{array}$$

Then the final trajectory feature a hidden state *feature*_*seq*_ is obtained by the weighted summation of the feature sequence as shown in Equation 25.


(25) $$\begin{array}{l} feature_{seq}=\sum_{i=1}^{T-k+1}\alpha_{i}\cdot feature_{st_{i}} \end{array}$$

Finally, we input the trajectory hidden feature states by multiple fully connected layers (Kingma and Ba, 2014; Zhang and Yang, 2018) (in the experiment, the hidden state dimension of the fully connected layer is 128) connected by the residual network structure. Recall that the residual network can avoid model degradation caused by depth deepening. At the tail of the residual network, we generate the collective prediction time *t*_*collective*_ by adding a fully connected layer with output dimension 1.

### 4.5 Model training

Finally, we will introduce the training steps of the model. We train an end-to-end model. In the training process of the model, we use the MAPE as our loss function and calculate the loss for individual and collective prediction simultaneously.

We specify a hyperparameter weight β between [0,1] and calculate the loss function value for individual prediction time and collective prediction time (Wang D. et al., 2018), respectively. Furthermore, weigh it using that hyperparameter weight β, balancing the individual and collective predictions, as described below.

We take a loss on the individual prediction as shown in Equation 26:


(26) $$\begin{array}{l} \;L_{individual}=\frac{1}{|T|\;-k\;+\;1}\\ \sum_{i=1}^{T-k+1}\frac{\left|t_{i}-\left(p_{i+k-1}.ts-p_{i}.ts\right)\right|}{p_{i+k-1}.ts-p_{i}.ts+\varepsilon } \end{array}$$

where ε is a small constant value that may approach zero in some exceptional cases due to the usually short time interval between two sampling points, and ε prevents the denominator from being equal to 0.

Take a loss on the collective prediction as shown in Equation 27:


(27) $$\begin{array}{l} L_{collective}=\frac{\left|t_{collective}-\left(p_{\left|T\right|}.ts-p_{1}.ts\right)\right|}{p_{\left|T\right|}.ts-p_{1}.ts} \end{array}$$

Our model will combine the above two losses as the final loss of the model prediction. By the hyperparameter β, our model can find the optimal solution by balancing between collective prediction and individual prediction as shown in Equation 28.


(28) $$\begin{array}{l} \beta \;\cdot L_{collective}+\left(1-\beta \right)\;\cdot L_{individual} \end{array}$$

During the testing of the model, our model predicts the collective travel time of the complete trajectory.

## 5 Experiment

In this section, we will describe the process of our experiment. We first test our model and other benchmark models in a realistic large-scale traffic dataset. Subsequently, we performed relevant experiments on the validity of the individual structures of our model.

### 5.1 Dataset setting

In this section, we will give a detailed description of the dataset and the preprocessing of the dataset.

#### 5.1.1 Dataset description

Chengdu Dataset: Chengdu Dataset (Wang D. et al., 2018) contained more than 9,737,557 trajectories (14 million GPS coordinates) for 14,864 taxis in Chengdu in August 2014. We remove some abnormal trajectories in the experimental process, such as unnatural driving speed and short driving distance.

#### 5.1.2 Data preprocessing

The Chengdu Dataset data set is divided into a plurality of data set files according to the specific date of the sampling day. The original format of each file is a path formed by a majority of GPS sampling points, and each path comprises a plurality of sampling points. The external information of each sampling point includes the route number, taxi number, latitude, longitude, passenger status (1 means carrying passengers, 0 means no passengers), and sampling time point. Examples of data are provided in Table 1.

**Table 1:** Data points.

1,300,30.4996330000,103.9771760000,1,2014/08/03/08:00:00

By identifying the route number and the driver number, we extract all the trajectory points of the same route, sort them according to the sampling time of the sampling points, and sort them into longitude and latitude sequences and passenger carrying state sequences. According to the start time of the trajectories, we calculate the sampling time sequence and calculate the travel time of the whole route according to the start and end time of the sampling of the sequence. The sequence of trajectories is shown in Table 2.

**Table 2:** Data description.

Trajectory number	1
Driver number	300
Weather status	Sunny, 16
Time	14:02
Longitude sequence	30.4996, 30.4886…
Latitude sequence	103.97717, 103.97727…
Sampling time series	0.0, 6.0…
Travel time	3096

In addition, considering the road properties of the city and the way the data set is taken, we exclude trajectories with a distance >100 km or < 0.5 km, a speed >100 km/h or < 5 km/h, and a time >7,200 s or < 60 s.

### 5.2 Parameter setting

In this section, we give some hyperparameter settings for our experiment.

1. Local Spatial Correlation Extraction Module

We set the dimension C of the learnable matrix $W_{local}\in R^{T\times C}$ to be 16-dimensional, so that the learnable matrix of the one-dimensional convolutional neural network, ${W_{conv}\in R}^{k\times C}$.

2. Spatial Correlation and Temporal Dependencies Extraction Module

We set the size of the *Q*, *K*, and *V* matrices in the Attention process in the global spatial correlation extraction module to (*T*−*k*+1) × *C*, where *T*−*k*+1 represents the length of the sequence and *C* is set to 16. In the attributes fusion process, the recurrent neural network part has its input dimension set to *C* + *E* and its output dimension set to *C*.

3. Multi-task Learning Module

We set the dimension of the hidden state of the residual network part of the multi-task learning module to 128. We set the superposition module of multiple linear layers to the decreasing term of dimension *C*, that is, 16 dimensions, 8 dimensions, and 1 dimension. We choose the hyperparameter β to be 0.3.

4. Hyperparameter Setting

We trained the model on the dataset 50 times for each experiment, and each experiment used the training parameters of the Adam optimizer, which has a learning rate of 0.001. The convolution kernel size of the local spatial feature extraction module is set to 3, and the β value of the multi-task learning module is set to 0.3.

### 5.3 Experiment environment

Our model is written using pyTorch, a deep-learning framework. We train/predict the model on an NVIDIA GeForce RTX 2080 Ti GPU, the CPU is Intel Core i7-9700F. We use the Adam optimizer (Kingma and Ba, 2014), the learning rate is set to 0.001, and the batch size is 32.

### 5.4 Experiment description

1. We use the first 20 days of the data set as the training set, the next 3 days as the validation set, and the last 7 days as the test set to test the predictive ability of the model under the full period (one week) training.
2. To determine the prediction ability of the model in a shorter period, we conduct experiments on a data set for 24 h and a week. First of all, we set the period of the data set to 24 h. We use the data sets of two adjacent days as the training set and the test set, respectively. To alleviate the interference of artificial time planning (such as weekends, holidays, etc.), we exclude the data sets of weekends, Mondays, and Fridays and take Tuesday as the training set and Wednesday as the test set. Moreover, in the attribute fusion module of the model, we exclude the date attribute of the target trajectory. Second, we select all the data sets of the previous week as the training set and Wednesday of the current week as the test set. We test our model on the above data sets.
3. To test whether the prediction ability of the model for arrival time will degrade with the increase of the time interval between training and prediction, we use the first week data set of Chengdu in August as the training set and the last week data set as the test set for experiments.
4. We conducted ablation experiments on each module of the model to determine the effectiveness of our proposed module.

### 5.5 Evaluation metric

We use mean absolute error (MAE), MAPE, and root mean square error (RMSE) as the evaluation matrix of our experiment.

MAE represents the mean of the absolute error between the predicted and observed values (Goodwin and Lawton, 1999).

MAPE is a relative measure, which is usually used to evaluate the prediction results of time series (Willmott and Matsuura, 2005).

RMSE is the square root of the ratio of the square of the deviation between the predicted value and the label value to the number of observations n, which measures the deviation between the predicted value and the label value (Chai and Draxler, 2014).

### 5.6 Performance comparison

In this section, we will compare the predictions of our model with those of other models.

#### 5.6.1 Baseline model

- GBDT: GBDT is an iterative decision tree algorithm. In the context of travel time prediction based on GPS trajectories, it combines the results of multiple decision trees to obtain the travel time. Since the length of GPS trajectories in this study is variable, and GBDT cannot directly handle sequences with variable lengths, this study uniformly samples each trajectory to a fixed length of 128.
- MLP-LSTM: We use a multilayer perceptron (five layers) and a two-layer LSTM-connected model as one of our baseline models, the input of the model is consistent with our model input, and the hidden layer size of the model is 128.
- WDR: The WDR model is a deep learning model that defines travel time prediction as a pure regression problem. It is primarily based on the wide–deep–recurrent framework, which learns different features of trajectories to accomplish travel time prediction.
- Deep TTE: The deep TTE model is currently one of the most accurate models for the time of arrival prediction based on GPS trajectory data sets. In this model, the geographic convolution module and the multi-task learning module based on attribute fusion are proposed for the first time. By extracting spatial and temporal features and balancing the global and local prediction capabilities of the model, the predicted values with high accuracy are obtained. However, in the module of spatial feature extraction, deep TTE only considers the spatial features of several adjacent path nodes and does not establish correlations between the global spatial features. It does not consider the change of attributes over time in attribute fusion. During the experiment, we set the dimension of its hidden state to be equal to our model.

#### 5.6.2 Results comparison

##### 5.6.2.1 Predictions on the full data set

We take the first 3 weeks of the data set as the training set and the last week as the test set and compare our model with the above model. As shown in Table 3, we can see that our model has achieved better prediction results compared with the previous method.

**Table 3:** Summary of experimental results.

**Evaluation metric**	**GBDT**	**MLP-LSTM**	**WDR**	**Deep TTE**	**AttentionTTE**
**1. Full dataset**					
MAPE	23.19%	18.73%	15.91%	14.10%	13.65%
MAE	391.42	387.13	307.42	227.38	227.38
RMSE	597.43	613.43	545.61	502.93	560.26
**2. Short period A**					
MAPE	12.49%	9.16%	9.32%	7.91%	6.84%
MAE	293.41	276.32	252.71	183.31	165.67
RMSE	587.81	523.71	545.27	399.83	386.83
**3. Short period B**					
MAPE	11.89%	8.21%	7.41%	5.41%	3.45%
MAE	262.73	166.62	128.51	105.87	74.38
RMSE	521.67	383.38	307.73	250.83	193.42
**4. Long time span**					
MAPE	25.36%	22.32%	23.71%	18.79%	17.95%
MAE	507.59	421.31	458.59	270.94	229.71
RMSE	927.41	948.02	937.31	576.56	553.67

##### 5.6.2.2 Predictive ability of the model on short-period data sets

For the training dataset with a period of 24 h, this study extracts Tuesdays and Wednesdays of all weeks in August from the complete dataset, selects Tuesday of each week as the training set of the dataset, Wednesday as the test set, and finally obtains four short-term datasets. This study tests the model of this article on these datasets.

For the dataset with 1 week, we take the trajectory data of the first 3 weeks (that is, 1 August to 22 August) in the dataset in August in Chengdu as the three training sets, and the trajectory data within the 3 weeks of the dataset in the last 3 weeks (that is, 8 August to 29 August) as the three corresponding test sets. Therefore, the period of the training set is an entire week. It can be seen from the experimental results that the model trained on the test set with a shorter period has higher prediction accuracy.

In addition, all models have significantly improved prediction accuracy on test sets over short time spans, so the current traffic state of a city may depend on the previous week's traffic status.

##### 5.6.2.3 Predictive ability of the model over a longer time span

We take the data set of the first week of August in Chengdu, from 4 August 2014 (Monday) to 10 August 2014 (Sunday), as the training set, and the data set of the last week as the test set for the experiment, from 25 August 2014 (Monday) to 31 August 2014 (Sunday), with a time span of 3 weeks. The data set on 3 August was used as a validation set to test the impact on the predictive ability model over a longer time span.

From the experimental results, the prediction ability of the model on the test set with a longer time span from the training set has degraded. Compared to the results of the previous experiment, this result may be because the current traffic state of the city is similar to that of the near time.

##### 5.6.2.4 Ablation experiment

During the ablation experiment, to reduce the training time of the model, we used the data sets from 3 August to 9 August as the training set and the data sets from 14 August and 15 August as the test set. The experimental results are described in Table 4.

a) Global Spatial Correlation Extraction Module
   We first train the model with or without using the global spatial correlations extraction module. The experimental results show that the results of the model using the global spatial correlations extraction module and the model without it on the above data sets are 4.09 and 5.79%, respectively, when MAPE is used as the evaluation matrix.
   We conduct further experiments on the Attention mechanism we proposed to verify that it is more efficient in revealing the correlations between the nodes of the sequence compared with the simple self-attention mechanism, which has the same hidden state dimension. The experiment results show that when using MAPE as the evaluation matrix, the model with the Attention module and the model with the simple self-attention module are: 4.09 and 4.53%. Experiments demonstrate the effectiveness of our model.
   During the process of integrating attribute features into the spatial-temporal features of trajectories, we conducted ablation experiments to investigate the fusion of attributes. We explored two approaches: directly concatenating the attribute feature vector with the spatial features of trajectories and using LSTM for attribute fusion. The experimental results are 4.09 and 4.17%, respectively.
b) Attribute fusion module
   To verify the effectiveness of the attribute fusion module, we conduct experiments on our model using the weighted attribute fusion module and our model using only the embedding coding technique. The experimental results show that, when MAPE is used as the evaluation metric, the results of the model using the weighted attribute fusion module and the model using only embedding coding attribute fusion module on the above data sets are 4.09 and 4.29%, respectively.
c) Multi-task learning module
   To verify the validity of the weighting method and to sum the spatio-temporal feature sequence by the attention matrix to obtain the features of the travel time of the complete path, we compare the experimental results of using the average method to get the travel time of the complete path. The results of the experiment show that the results of the model using the weighted summation of the attention matrix and the model using the averaging method on the above data sets are 4.09 and 4.21%, respectively, when MAPE is used as the evaluation metric.
d) Model size
   The trainable parameter size of our whole model is about 0.56 MB. The trainable parameter of the weighted attribute fusion module is about 0.21 MB, the trainable parameter of the spatial correlations and temporal dependencies extraction module (including the local spatial correlation extraction module) is about 0.27 MB, and the trainable parameters of the multi-task learning module is about 0.08 MB. Compared with DeepTTE, a deep learning model with higher prediction accuracy (the trainable parameters of the model are about 0.71 MB, and the attribute fusion module is about 0.40 MB), there are fewer trainable parameters. The model has fewer trainable parameters because the encoding latitude of each attribute in the attribute embedding process is limited to a lower value.

**Table 4:** Summary of ablation experimental results.

**Model**	**MAPE**	**MAE**	**MSE**
No global spatial correlation extraction module	5.79%	109.32	233.78
No attribute fusion module	4.29%	102.45	197.43
No multi-task learning module	4.21%	112.68	201.54
AttentionTTE	4.09%	97.85	191.41

## 6 Conclusion and future work

In conclusion, we propose a novel external information fusion method, the weighted attribute fusion module, which assesses the impact of various attributes, such as driver information, on prediction results in different scenarios. This innovative approach significantly enhances the accuracy of travel time estimation by integrating both global and local spatial correlations with temporal dependencies. Looking ahead, the AttentionTTE model holds significant potential for future urban projects, particularly in the areas of electric vehicle (EV) scheduling (Ochoa and Oliva, 2018) and the transition from conventional vehicles to EVs (Ghasemlou et al., 2023). Accurate ETA predictions are crucial for optimizing EV charging schedules and ensuring efficient route planning, which can reduce energy consumption and improve overall transportation efficiency. As cities continue to evolve toward sustainable and intelligent transportation systems, integrating the advanced predictive capabilities of AttentionTT can facilitate smoother and more effective transitions in urban mobility infrastructures.

## Data availability statement

The original contributions presented in the study are included in the article/supplementary material, further inquiries can be directed to the corresponding authors.

## Author contributions

ML: Writing – original draft, Writing – review & editing. YF: Writing – review & editing. XW: Writing – original draft.
